# Understanding Adolescent Anti‐Retroviral Therapy Use in the BREATHER‐Plus Trial: Qualitative Findings From South Africa and Uganda

**DOI:** 10.1002/jia2.70207

**Published:** 2026-09-22

**Authors:** Londiwe Shandu, Stella Namukwaya, Tamlyn Carmin Seunanden, Nothando Ngwenya, Moherndran Archary, Cissy Kityo, Deborah Ford, Sarah L. Pett, Adeodata R. Kekitiinwa, Sarah Bernays, Janet Seeley, the BREATHER‐Plus trial team

**Affiliations:** ^1^ Africa Health Research Institute KwaZulu‐Natal South Africa; ^2^ MRC/UVRI and LSHTM Uganda Research Unit Entebbe Uganda; ^3^ School of Nursing and Public Health University of KwaZulu‐Natal Durban South Africa; ^4^ Department of Psychiatry University of Oxford Oxford UK; ^5^ Enhancing Care Foundation, Department of Paediatrics and Children Health Victoria Mxenge Hospital University of KwaZulu‐Natal Durban South Africa; ^6^ Joint Clinical Research Centre Kampala Uganda; ^7^ Medical Research Council Clinical Trials Unit at University College London (UCL) London UK; ^8^ Baylor College of Medicine Children's Foundation Kampala Uganda; ^9^ Sydney School of Public Health University of Sydney Sydney New South Wales Australia; ^10^ Department of Global Health and Development London School of Hygiene & Tropical Medicine London UK

**Keywords:** adolescents, antiretroviral therapy, clinical trials, HIV, South Africa, Uganda, young people

## Abstract

**Introduction:**

The BREATHER‐Plus trial was an open‐label, randomized non‐inferiority trial comparing weekends off antiretroviral therapy (ART) (short cycle therapy [SCT]) with daily oral ART (continuous therapy [CT]) for adolescents aged 12–19 years. We present findings from the qualitative sub‐study on the factors influencing trial participants’ decisions to take/not take ART in accordance with their randomized arm.

**Methods:**

Qualitative data collection was conducted in South Africa and Uganda from December 2022 to July 2024 using in‐depth interviews (IDIs) and focus group discussions (FGDs). We purposively recruited 29 trial participants (14 SCT/15 CT arm) to take part in IDIs in South Africa. From the trial sites in both countries, 47 trial participants, 17 non‐trial participants, 15 caregivers and 16 healthcare providers took part in FGDs. Data collection with trial participants focused on adherence to SCT compared to daily ART; the acceptability of SCT and the experience of taking part in the trial. All FGDs explored the feasibility of SCT and preferences for alternative approaches to accessing therapy. Data were analysed thematically.

**Results:**

Both IDI and FGD data showed that the opportunity to have weekends off from treatment was welcomed by participants, caregivers and healthcare providers. However, the understanding of “2 days off” treatment was interpreted flexibly by some participants. Trial participants in both arms described their fluctuating adherence practices both prior to and during the trial. The introduction of SCT as a possibility through the trial prompted some to talk about previously concealed missed doses. Participants reported that they only mentioned missed doses in the clinic if they had a raised viral load result or had returned to the clinic with too many pills. Many participants relied on their viral load result to confirm that non‐adherent behaviour was not problematic.

**Conclusions:**

Our findings offer insights into the factors that shape ART adherence among adolescents living with HIV. Adherence is not a static behaviour but a socially embedded and relational practice, shaped by adolescents’ interpretations of treatment, interpersonal relationships and the structure of care environments. Regimen modification alone does not resolve the broader social and emotional challenges adolescents face.

## 1 Introduction

There has long been a quest to find ways to lessen the burden on people living with HIV who, with the current oral regimens, need to take antiretroviral therapy (ART) tablets every day and lifelong [1, 2]. Several studies and randomized trials in adults have explored 2–3 days off ART at the same time during the week, with encouraging results. However, these trials have been conducted predominantly in adults in high‐income settings, and with very frequent viral load monitoring [3, 4].

Addressing threats to adolescents’ HIV treatment engagement (that is suboptimal adherence to treatment and high rates of attrition from care [5]), has prompted exploration of various treatment interventions. The safety and effectiveness of using treatment breaks from oral therapies to support adolescents’ adherence was investigated in the BREATHER (BREaks in Adolescent and child Therapy using Efavirenz and two nRtis [Nucleoside/nucleotide Reverse Transcriptase Inhibitor]) PENTA 16 trial (2011−2015). The findings published in 2016 showed that a short‐cycle oral ART strategy was a viable option for young people living with HIV who were adherent to their ART regimen [6, 7]. The findings demonstrated the non‐inferiority of short cycle therapy (SCT) versus continuous therapy (CT) at 48 weeks, by maintaining virological suppression in children, adolescents and young adults in the trial [8]. However, this investigational dosing strategy was not adopted as a standard treatment approach for adolescents and adults. The findings from the qualitative component of the BREATHER trial, published in 2017 [9], highlighted that non‐disclosure of adherence behaviours which did not follow the SCT regimen was not uncommon, with young people interpreting a non‐detectable viral load revealed during clinic‐based tests as demonstrating that recent missed doses were not significant. In a final workshop with the participants in the qualitative sub‐study, many expressed the fear that rolling SCT out to others who were not as successful as they were in managing their condition could pose significant risks to those adolescents and to the wider population living with HIV, should drug resistance increase as a result [9]. Indeed, in all the trials with adults where SCT was used, the investigators have stressed the need for exemplary adherence to the regimen to ensure success (see, e.g. 2, 10, 11, 12). Our analysis in this paper builds on this to examine how adolescents understood, interpreted and implemented SCT within the context of the BREATHER‐Plus trial.

The BREATHER‐Plus trial (2022−2025) was an open‐label, randomized (1:1) non‐inferiority trial comparing SCT with CT for adolescents using tenofovir disoproxil fumarate/lamivudine/dolutegravir. Four hundred and seventy adolescents living with HIV (ALHIV) aged 12 to <20 years were recruited from Kenya, South Africa, Uganda and Zimbabwe. As 10% versus 5% of those in the SCT versus CT arm, respectively, had confirmed viral loads ≥50 copies/mL, the strategy of SCT cannot be recommended, especially with the level of standard‐of‐care viral load monitoring (at best 6‐monthly in the trial settings) [13]. In this paper, we present the primary study findings, by study arm, from the qualitative sub‐study of the BREATHER‐Plus trial to describe how ALHIV, together with their caregivers and healthcare providers (HCPs), understood study procedures and the monitoring of their adherence to either SCT or CT, and how the adolescents reconciled protocol requirements with the realities of their day‐to‐day lives.

## 2 Methods

### 2.1 Setting and Study Design

The qualitative component of the BREATHER‐Plus trial was conducted in South Africa and Uganda, in three trial sites: the Durban International Clinical Research Site/Enhancing Care Foundation in eThekwini, South Africa and Baylor College of Medicine Children's Foundation and Joint Clinical Research Centre, both in Kampala, Uganda. Data were collected from December 2022 to July 2024 using IDIs and FGDs.

### 2.2 Study Participants and Sampling

ALHIV attending the clinical trial sites were eligible for the BREATHER‐Plus trial if they had been virologically suppressed for 12 months at enrolment, on ART for ≥ 1 year with no previous treatment failures and aware of their HIV status. Adolescents enrolled in the trial were randomized to one of two arms: SCT, where they took ART for 5 days and had 2 days off therapy at the weekend, or CT, where they took ART every day (standard of care). Inclusion in the qualitative study required them to be willing to disclose information about their HIV status and their ART‐taking to the research team.

**FIGURE 1:**
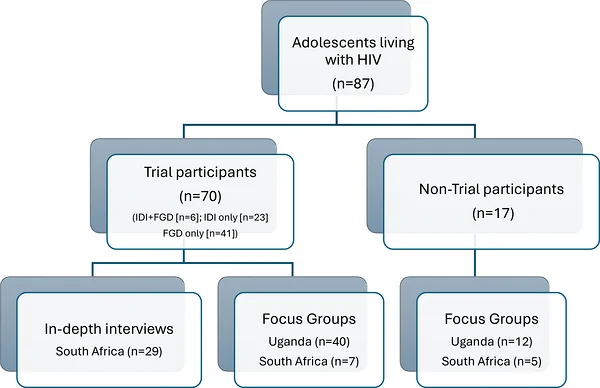
The sample for the qualitative sub‐study participants.

We purposively recruited 88 adolescents to take part in the qualitative sub‐study, and refer to them as participants, differentiating those who were in the trial (trial participants) and those who were receiving HIV care from the clinical trial sites but not in the trial (non‐trial participants). Participants were stratified according to age, gender and trial arm to ensure representation of these characteristics and to capture diverse experiences and perspectives. Twenty‐nine trial participants out of 30 selected took part in one‐to‐one IDIs in South Africa (see Table 1). Forty trial participants in Uganda and seven from South Africa also took part in FGDs; six of the seven FGD trial participants in South Africa, at their request, also took part in IDIs because they wanted to share their own individual experiences. In addition, 17 non‐trial participants, adolescents living with HIV who were attending the same clinic as trial participants but not taking part in BREATHER‐Plus (five from South Africa and 12 from Uganda), took part in FGDs. Fifteen caregivers (12 female, three male) and 16 HCPs from the trial sites in both countries also took part in separate FGDs (see Figure 1 and Table 2).

**TABLE 1:** In‐depth interview of BREATHER‐Plus trial participants in South Africa.

Interview	No	Age range	Female	Male	SCT	CT
1	29	12−19	15	14	14	15
2	27	13−19	14	13	13	14

**TABLE 2:** Focus group discussion participants in BREATHER‐Plus South Africa and Uganda.

Participant group	Site	Age range (years)	No of FGDs	Female	Male	SCT	CT
**BREATHER‐Plus Trial**	SA	18−19	1	4	0	4	0
		14−17	1	0	3	3	0
	UG	18−19	2	6	0	6	0
				0	6	6	0
		15−17	2	6	0	6	0
				0	5	5	0
		15−19	1	5	0	0	5
		12−14	2	6	0	0	6
				0	6	6	0
**Non‐trial adolescents living with HIV**	SA	18−19	1	3	2	N/A	N/A
	UG	15−19	2	6	0	N/A	N/A
				0	6	N/A	N/A
**Healthcare providers at study clinics**	SA	30−60	1	11	1	N/A	N/A
	UG	30−50	1	2	2	N/A	N/A
**Caregivers of participants in BREATHER‐Plus**	SA	42−59	1	4	0	N/A	N/A
	UG	30−60	2	11	0	N/A	N/A

All the participants taking part in this sub‐study had acquired HIV at birth. The majority had been taking ART for more than 10 years. However, five participants from South Africa were reported to have begun taking ART 3−9 years before the trial began.

### 2.3 Data Collection

In South Africa, data were collected using IDIs and FGDs, while in Uganda, data were only collected using FGDs. The IDIs and FGDs with trial participants focused on adherence to SCT compared to daily ART; the acceptability of the treatment strategy for participants in each arm and their experience of taking part in the trial.

In South Africa, two IDIs were conducted with each trial participant 6 months apart. We purposively selected 15 trial participants from each trial arm, stratified by sex and age. Twenty‐nine trial participants took part in the first round of interviews, and 27 in the second. Two participants were unavailable at the time of the second interview. An isiZulu (local language) speaking social scientist, LS, conducted the interviews.

FGDs were conducted with trial participants (split by arm), non‐trial clinic patients, caregivers and HCPs, in separate groups. These explored the feasibility of SCT and preferences for ways to access therapy. The focus groups were facilitated by LS and TCS in South Africa and SN, a Luganda (local language) speaking social scientist, in Uganda.

### 2.4 Data Management and Analysis

Interviews and FGDs were digitally recorded, transcribed and translated. For the analysis for this paper, themes were drawn from both the topic guide: experience of participating (or not participating) in the trial, treatment adherence, and observations on help and support received for those in the trial and from the data (communication). The first two authors read and re‐read transcripts and then coded the data before discussion on the emerging analysis with JS. After coding, analytical memos by theme were prepared and shared by the first and second authors with JS, SB and NN and discussed and refined before reworking into the findings presented here.

The qualitative data are stored on a secure computer server at each study site and only authorized study staff have access to this information. These data are managed following site‐specific policies aligned with GDPR/Protection of Personal Information (POPI) regulations.

All quotes are labelled with age (for adolescents) gender, trial arm (SCT/CT) or non‐trial participant (NTP), caregiver or HCP, data collection mode (IDI/FGD) and country, for example: (18‐year‐old female‐SCT‐FGD‐Uganda).

### 2.5 Ethical Considerations

For South Africa, ethical approval was obtained from the University of KwaZulu‐Natal (UKZN) Biomedical Research Ethics Committee (BREC Ref No: 4160/2022) and Pharma Ethics (210,624,036). In Uganda, approval was obtained from the Joint Clinical Research Centre Ethics Committee (JCRC 2021–19, 22 September 2022) and the Uganda National Council for Science and Technology (HS1822ES, 15 November 2021). BREATHER‐Plus is registered with PACTR, number 202103692694276; ISRCTN number 85058577 and CTA number 27505/0005/001‐0001. Written informed assent and consent were obtained from all participants, including minors and parents/legal guardians, before study participation.

## 3 Results

The findings from adolescents, caregivers and HCPs reveal how participants made sense of the treatment regimens, how they interpreted and adapted to the SCT regimen, and how adherence practices were negotiated within their social and relational environments. We also show how participants’ understanding of the trial and communication practices influenced their decisions to take or skip treatment, and how these behaviours were shaped by clinical feedback, peer and family support, and broader life circumstances.

### 3.1 Understanding the Purpose of the Trial

All the participants who were part of the SCT arm were enthusiastic about the prospect of some respite from daily pill‐taking and, in some cases, the hope that 1 day there would be further reductions in treatment frequency. Several spoke of their happiness when they found out they were on the SCT arm, as one participant from Uganda described: “*When they told me that I was going to have a break I was very happy. Now on weekends I can play until I get tired without having to worry about taking treatment*.” (15‐year‐old female‐SCT‐FGD3‐Uganda).

Some spoke of their fatigue with long‐term treatment. One participant said: “*when I heard that I could miss to take the medicine I was happy because I was tired of having to take my medicine*.” (14‐year‐old female‐SCT‐FGD12‐Uganda). For others, the trial raised hopes that 1 day they may not have to take treatment at all: “*BREATHER‐Plus is being done to see if the medicine that they are giving us every day can be taken on fewer days in the week as a way of giving us hope that we will get cured*.” (12‐year‐old male‐SCT‐FGD12‐Uganda). Another young woman said: “*It gave me hope, because if I can survive for 2 days without taking treatment then in future the days off treatment might be increased… who knows, we might even take treatment just once a week*.” (18‐year‐old female‐SCT‐FGD1‐Uganda). And one participant in South Africa expressed the wish that if he could take the treatment less frequently, then: “*Maybe there is something they can do to take this virus out of me*.” (17‐year‐old male‐SCT‐IDI‐South Africa).

The primary appeal of SCT centred on the freedom at weekends. One participant explained, “*The weekend is for partying… sometimes you may go out and during that time you will not move around with the medicine in the bag*.” (18‐year‐old female‐SCT‐FGD1‐Uganda). Not having to remember their drugs reduced the logistical constraints they usually faced, taking their ART at a set time each day. This was something the non‐trial participants also reflected on in Uganda: “*it(SCT) is good because it can reduce the burden of having to take the medicine. Most of my important jobs happen during the weekends so having a break would be good*.” (16‐year‐old‐male‐FGD3‐NTP‐Uganda).

In both countries, the participants spoke of the feeling of “normalcy” as they enjoyed going out at weekends with their peers unencumbered by the pressure of remembering to take their treatment, as well as not having to hide taking their medication from weekend visitors to their home or from friends when they were away for the weekend. This was something several caregivers reflected on, for example: “*My son plays sports and sometimes they go to camps, and having to carry medication with him to camp becomes an issue. A weekend break is better*.” (Caregiver‐FGD5‐South Africa).

Not surprisingly, most adolescents who had been randomized to be on CT wished they had been selected for the SCT arm. For some, the expectation of time off from treatment was the reason they agreed to join the trial: “*I read that in the study some people would have a break from taking their medication so I accepted because I thought that I would be one of those chosen to have a break*.” (15‐year‐old female‐CT‐FGD5‐Uganda). While several of the participants on the CT arm spoke of their disappointment, a few said they were relieved because they were fearful of what a break might do to the tolerance of their body to ART, although it may be that they were simply trying not to seem disappointed.

### 3.2 Flexible Adherence Practices Within Trial

Most participants had a good understanding of the purpose of the trial, framing it as testing whether adolescents living with HIV could safely take breaks from daily oral ART. One participant explained that the trial was: “*a study to see if a person can survive well if they have a break from taking ART*.” (17‐year‐old female‐SCT‐FGD3‐Uganda). For several trial participants, their understanding of the trial translated into its strict application in practice: “*SCT is when we are allowed to take a break, but we have to follow instructions carefully*.” (12‐year‐old male‐SCT‐FGD12‐Uganda). However, this precision was not followed by all trial participants, and it was clear from the way in which some trial participants managed their pill‐taking in the trial, that the 5 days on, 2 days off, was sometimes interpreted to be more flexible than intended. Indeed, several participants indicated that they were not adhering strictly to the prescribed breaks at weekends: “*[…] sometimes I do not clearly remember whether today I'm supposed to take them or stop. Sometimes when I remember, I would have already taken pills for too many days*.” (17‐year‐old male‐SCT‐IDI‐South Africa). Some believed they could choose any 2 days to rest, as an 18‐year‐old young woman in Uganda explained: “*They told us to choose 2 days off (for SCT) medication during the week… yes, 2 days of our choice*.” (18‐year‐old female‐SCT‐R6‐FGD1‐Uganda). This meant that some trial participants adjusted the schedule for personal convenience, skipping different days or substituting days off: “*when we go out on Fridays to have fun, I don't take the pill. I just make up for it over the weekend*.” (17‐year‐old male‐SCT‐IDI‐South Africa). However, others misunderstood the duration or frequency of skipped doses: “*I thought since we rest on weekends, if I forget during the week, it is also okay*.” (18‐year‐old male‐SCT‐R2‐FGD2‐Uganda). A few confided that this misunderstanding led to more dramatic slippages in treatment taking: “*I thought SCT means I can take medicine only when I feel like it or when I remember*.” (19‐year‐old male‐SCT‐FGD2‐Uganda).

Reflecting a potentially broader pattern about the inconsistent execution of SCT, there were also some in the continuous arm who alluded to the likelihood of their own non‐compliant adherence behaviour should they have been allocated to the SCT arm. For example, one of the Ugandan participants in the CT arm, when asked how he would have managed SCT, said that he would have had 3 days off, not just 2, adding that he thought that is what people on the SCT arm would be doing.

During analysis, we compared adherence‐related experiences and patterns across male and female participants and did not find gendered differences in the data from the sub‐study on how adolescents described adherence behaviours.

### 3.3 “Testing” What Needs to be Said

Trial participants in both arms described their fluctuating adherence practices both prior to and during the trial. “*I have missed to take my medicine many times because I am so tired of taking it*.” (13‐year‐old male‐SCT‐R5‐FGD12‐Uganda). Some caregivers expressed their sympathy for their children who were weary of ART: “*Even for us as adults, this is tiring. You can see that taking pills is burdensome, but we have no choice*.” (Caregiver‐FGD5‐South Africa). Trial participants explicitly admitting missed doses was rare though, and talk about how often they missed doses was commonly couched in ambiguous terms: “*I used to forget sometimes. If I was on the one where you rest on Saturday and Sunday, maybe I would be better*.” (16‐year‐old male‐SCT‐IDI‐South Africa).

The introduction of SCT as a possibility through the trial appeared to open up the opportunity for adolescents to talk about otherwise previously concealed missed doses: “*Even before the study there are times when I would spend two days a week without taking my treatment*.” (15‐year‐old female‐CT‐FGD5‐Uganda). Their overall hesitation to discuss their adherence challenges demonstrated both an uncertainty in how such admissions would be received by others and the relational constitution of adherence practices: “*I take the pill when my mother reminds me, if she forgets then I also forget*.” (14‐year‐old male‐SCT‐IDI‐South Africa). In the discussions with healthcare workers, it was clear they understood the challenges young people faced: “*I have dealt with young people for a long time, and they really get tired of the medicine. They even tell you that*, ‘*doctor, I am tired of the medicine*’.” (Female‐HCP‐FGD‐Uganda).

Many trial participants subtly conveyed a pattern of missed doses in their interactions with the team in the qualitative sub‐study, particularly in South Africa in the more intimate setting of a one‐to‐one IDI with the interviewer. Trial participants reflected that, although missed doses were discussed tentatively in these qualitative interactions, most would be reluctant to discuss their adherence practices with comparable candour in clinic conversations. Instead, trial participants described the efforts they invested in avoiding “difficult” conversations with both family members and particularly clinic staff. For example, concealing any unused pills so as not to be “discovered”: “*I usually hide my pills in the bucket where they would not see them*.” (16‐year‐old male‐CT‐IDI‐South Africa). Another trial participant commented: “*Oh, what I do is that I sometimes go and count them. I took the extra ones and set them aside. I took out the extra ones, the ones that I was supposed to take and show them the correct ones*.” (16‐year‐old male‐SCT‐IDI‐South Africa). Others admitted that this concealment was done with the help of relatives to avoid getting into trouble when attending the clinic: “*The pharmacist does the pill count so I count them before going there and mom would take out the others and keep them at home*.” (14‐year‐old male‐SCT‐IDI‐South Africa).

A common pattern across the data was that many adolescents were “scared to say” about slippages in adherence behaviour: “*For me I did not tell them… I was afraid they would quarrel*.” (18‐year‐old female‐SCT‐FGD 1‐Uganda). Often this fear was anticipatory rather than based on experience, as the same participant clarified, “*that's just my way of thinking because usually when we don't follow instructions even at home that is what happens*.”

Adolescents were clear, however, that they tended to only “talk” about missed doses in their clinic visits, if they were “caught” either through returning with too many pills, or by their behaviour showing in a raised viral load result. “*When I came to the clinic, I told the nurse (about missing doses) because he counted my pills, and there were more than what they should have been. But if he had not noticed it, I would not have told him*.” (12‐year‐old male‐SCT‐R3‐FGD12‐Uganda). Adolescents explained how they assumed that their ambiguous communication about adherence practices was permissible because any negative effects of missed doses were presumed to be captured within viral load test results. If viral suppression was maintained, flexible adherence was interpreted as acceptable and treated as a licence to continue their practice (of missed doses) “safely.” As one trial participant said, “*When the doctor said my blood was fine, I thought even if I miss, I am still okay*.” (18‐year‐old male‐SCT‐FGD2‐Uganda).

This ambiguous depiction of adherence practices was recognized by HCPs who acknowledged that some adolescents deliberately obscured their adherence behaviour. They described SCT as offering some relief from treatment fatigue, which they knew characterized adolescents’ treatment experience. But some also considered it as a mechanism to make official the skipped doses that they anticipated many adolescents were doing anyway: “*Sometimes, the patients give themselves holidays so if this is official and the study was done on it, I felt that it would be something good for the young people*.” (HCP‐R4‐FGD4‐Uganda).

In common with adolescents, HCPs similarly articulated a reliance on viral load test results to “expose” such behaviour: “*They even hide pills at home, so it looks like adherence is 100%, but the bloods say otherwise. They have many tricks, but the blood results expose them*.” (HCP‐R1‐FGD4‐Uganda).

For many adolescents, the confidence invested in the “truth‐telling” of viral load test results meant that for some it became a substitute for more open discussion. This did not apply to all participants, though, with some adolescents emphasizing that if they did miss, they always told clinic staff because, “*you have to tell them the truth always*.” (17‐year‐old female‐SCT‐FGD3‐Uganda).

Despite this complex communication environment, in which adolescents had varying expectations about the need for, and the effect of, talking candidly about missed doses, participants almost consistently emphasized the valuable support they received from clinic staff. Trial participants described the trial as a setting that offered solid, dependable support which directly influenced their motivation and ability to adhere to treatment. “*Every time I come to the clinic, they encourage me and motivate me when they tell me that my virus is sleeping*.” (14‐year‐old male‐SCT‐FGD12‐Uganda). They cited the care that they received as being instrumental in them maintaining their engagement in care, as one participant shared, the research site felt like “*a good place… it helps many people*.” (18‐year‐old male‐CT‐IDI‐South Africa).

### 3.4 Relational Influences on Adherence Practices

It was evident from participants’ accounts that adherence is a relational practice. Before the trial, adolescents described a range of supportive structures that shaped their early adherence habits. Parents were central in establishing these routines, often serving as the main source of daily reminders and guidance: “*I grew up with my mother telling me to take the pills*.” (18‐year‐old female‐SCT‐FGD1‐Uganda). When parental supervision was limited due to work or other responsibilities, other family members, particularly grandparents, stepped in to provide consistent oversight. As illustrated above caregivers were also, at times, involved in concealing missed doses to protect reputational capital and avoid potential repercussions at the clinic.

The influence of caregivers on adherence practices tapered as adolescents grew older, with peers becoming increasingly influential, which could prompt additional disruptions to treatment taking. “*During the holidays when I go away from home, I don't take the medicine because I don't want others to see me*.” (18‐year‐old male‐CT‐FGD6‐Uganda).

Alongside mitigating the “fatigue” of continuously having to take pills, adolescents of all ages talked about the strategic advantage of the SCT regime in supporting the concealment of their HIV status from others: “*at home, every weekend we get visitors yet taking the medicine is a secret between me and my mother…I joined the study to have a break on the weekend while we have visitors at home*.” (12‐year‐old male‐SCT‐FGD12‐Uganda).

Among the older adolescents who admitted to being in romantic relationships, the opportunity to avoid taking treatment at the weekends was cited as an incentive to participate in the trial because it reduced treatment‐related stress through lowering the risk of accidental discovery and unintended disclosure. Trial participants described how deductive disclosure was a continuous source of worry: “*Some boyfriends can check your bag once you are not there, and they may find your medicine*.” (18‐year‐old female‐SCT‐FGD1‐Uganda). A few of the older trial participants interviewed in South Africa, who talked about their relationships, described how their knowledge of the protective effects of viral suppression on onward transmission encouraged them to adhere carefully to the 5 days on and 2 days off schedule.

Our findings offer insights into the factors that shape ART adherence among adolescents living with HIV. Participants’ engagement with the SCT regimen was influenced not only by clinical guidance but also by their understanding of the study, their interpretation of viral load test results and relationships with peers and caregivers, and their daily routines. Decisions to take or miss doses reflected a continuous balancing of medical advice, social expectations and individual circumstances, which was moderated by complex communication practices, highlighting the interactions between knowledge, support and lived experience.

## 4 Discussion

The qualitative component of the BREATHER‐Plus trial examined how adolescents living with HIV, their caregivers, and HCPs understood and navigated ART in Uganda and South Africa. The findings demonstrate that adherence is not a static behaviour but a socially embedded and relational practice, shaped by adolescents’ interpretations of treatment, interpersonal relationships and the structure of care environments. These findings align with and extend the existing literature on adolescent HIV care by highlighting how biomedical innovation intersects with lived experience to support the management of HIV by adolescents living with the condition.

Consistent with prior research, adolescents in this study described ongoing challenges associated with taking daily oral ART, including treatment fatigue, disruption of routines and emotional exhaustion (e.g. 14, 15, 16, 17). Adolescence is widely recognized as a period marked by transitions, identity formation and heightened sensitivity to peer influence, all of which can complicate long‐term treatment adherence [18, 19]. Participants in this study did not frame non‐adherence as a rejection of treatment, but rather as a response to the cumulative burden of lifelong medication.

While most adolescents demonstrated a basic understanding of the 5 days on and 2 days off structure, some reinterpreted this flexibility to suit personal circumstances, with our findings indicating that some adolescents in the SCT arm were not consistently only taking 2 days off per week. Similarly, some participants in the continuous arm were also struggling to comply with their prescribed doses every day, with some expressing their disappointment of being on CT. This mirrors earlier findings from the BREATHER trial, where we noted that some participants reinterpreted treatment instructions to fit their schedules [9].

Clinical monitoring, particularly viral load feedback, played a powerful role in shaping adherence behaviour in the current study (which undertook less monitoring than other SCT trials). Adolescents often interpreted viral suppression as providing reassurance that their treatment practices were effective, while some participants equated an undetectable viral load with reduced vulnerability, occasionally justifying additional missed doses, as has been observed elsewhere [20, 21]. “Reassuring” viral load results became a substitute for ongoing conversations about adherence and medication‐taking behaviour. HCPs in this study recognized the need to contextualize viral load results carefully, supporting evidence that clinical feedback must be accompanied by a clear explanation to prevent misinterpretation [22]. This is an important learning to support future adherence to drug therapies.

Drug type has played an important role in all intermittent therapy trials. Trials have used combinations of recommended drugs, as the ART field has evolved to find better‐tolerated treatments. These combinations have included efavirenz (a non‐nucleoside reverse transcriptase inhibitor)‐based regimens [1, 3, 6]). Efavirenz is a drug with a prolonged half‐life (35−50 h) compared to dolutegravir, which has a half‐life of 13−14 h [23, 24]. Efavirenz has ceased to be a drug of first choice for ART because of its neuropsychiatric effects and low genetic barrier to resistance. Since 2019, dolutegravir has been recommended by the World Health Organization as the first‐ and second‐line treatment for people living with HIV [25]. A tenofovir disoproxil, lamivudine and dolutegravir regimen was used in the BREATHER‐Plus trial. It seems possible that the regimen, which included efavirenz, used in the first BREATHER trial allowed the adolescents taking part in that trial to skip additional doses without experiencing raised viral loads. The qualitative findings indicate that adolescents participating in the BREATHER‐Plus trial experienced, potentially, similar challenges in maintaining the necessary “exemplary adherence” to their regimen but without the same clinical effect. The BREATHER‐Plus trial results mean that while SCT offers an appealing option for adolescents using current first‐line therapy, it cannot currently be recommended for use in low‐ and middle‐income settings, as is stated in the paper presenting the trial findings [13].

### 4.1 Strengths and Limitations

A strength of this study is the detailed information that adolescents participating in a trial were able to share with the study team. While there is a wealth of data from the FGDs in Uganda, the more intimate setting of the IDIs in South Africa, and the longitudinal nature of that data collection (two interviews and engagement in an FGD) appears to have allowed greater disclosure of what could be considered “transgressions”: skipped doses and discarded pills. A limitation is that we do not have IDI data from Uganda; this was a decision based on the anticipated resources available during the planning for the trial as well as the knowledge that IDIs had been conducted in the BREATHER trial in Uganda, and this study was anticipated to build on those data. That said, the FGD data from Uganda are detailed and revealing, reflecting the willingness of the participants to share with a skilled facilitator.

## 5 Conclusions

The findings contribute to the literature on the experience and motivations of clinical‐trial participants, and highlight the importance of understanding participants’ adherence behaviour within trials to explain clinical outcomes. While SCT was largely welcomed, the findings indicate that regimen modification alone does not resolve the broader social and emotional challenges adolescents face. In response, adolescents in both trial arms actively negotiated treatment flexibility, informally modifying their prescribed regimens and testing boundaries based on social needs and clinical reassurance.

The adherence challenges adolescents experience can be considered relatively predictable and could be anticipated by HCPs and caregivers. Responding sympathetically will foster safe conversation spaces in clinical settings that encourage adolescents to be more open about their adherence practices. This would serve to strengthen the effectiveness of support and monitoring, which are vital to achieving optimal HIV outcomes.

In the wake of funding cuts to HIV programmes, the possibility that ART may not need to be taken every day had been seen as a way to stretch supplies [26]. Given the BREATHER‐Plus trial findings, hope lies for adolescents in future drug regimens including long‐acting oral combinations and long‐acting injectable combinations. These hold real promise of simplifying HIV treatment across the age spectrum and should be a priority area of research in low‐ and middle‐income settings.

## Author Contributions

LS and SN: Investigation, data curation, formal analysis and Writing – original draft. TCS: Investigator, data curation, Writing – review and editing. JS, NN and SB: Conceptualization, methodology, supervision, formal analysis, Writing – original draft, Writing – review and editing. MA and CK: Supervision, project administration, Writing – review and editing. DF, SLP and ARK: Funding acquisition, project administration, Writing – review and editing.

## Conflicts of Interest

All the authors declare that they have no conflicts of interest.

## Data Availability

The data that support the findings of this study are available on reasonable request through the Chief Investigator, ARK. The data are not publicly available due to privacy or ethical restrictions.

## BREATHER PLUS/LATA Consortium Members

**MRC CTU at UCL**: Sarah Pett, Deborah Ford, Margaret Thomason, Alasdair Bamford, Ellen White, Jessica Kirk, Angus Jennings, Helen Ainscough, Rebecca Dodds, Molly Bush, Simona Salomone, Hanh Nguyen, Paul Crawley, Alexandra Green, George Pettitt, Jemima Shickle, Stephen Townsend, Hannah Sweeney, Margaret Hook, Sara Peres, Nadine Van Looy, Andy Sum, Diana Gibb, Annabelle South, Elizabeth Chappell, Aoife Nolan

**Baylor College of Medicine Children's Foundation, Uganda**: Adeodata Kekitiinwa, George Patrick Akabwai, Fredrick Katongole, Naomi Apoto, Resty Babirye Okello, Ronald Nabimba, Muzamil Nsibuka Kisekka, Collins Mujyanama, Gerald Agaba Muzorah, Gideon Ahimbisibwe, Florence Namuli, Rose Namaganda, Lameck Kiyimba, Angella Baita, Rachael Namuddu Kikabi, Henry Balwa, Susan Tukamuhebwa, Maria Benita Aino, Judith Tikabibamu, Anthony Kirabira, Lekku Lawrence, Barbra Nantume, Muhammad Lutalo, Diana Louis Anena, Rose Jacqueline Kadhuba, Patricia Nahirya Ntege, Teddy Kembabazi

**Joint Clinical Research Centre, Uganda**: Cissy Kityo, Victor Musiime, Shamim Nakabuye, Miriam Namasinga, Peace Hellen Masiga, Roselyn Kabasingo, Claire Nasaazi, Centurio Wandera, Josephine Kobusingye, Disan Mulima, Alex V Musiime, Faith Mbasani, Ezra Lutalo, Brenda Namukwaya Efrance, Odoch Denis, Baliruno David, Ampaire Phionah, Edgar Ayesiga Wandigali, Patrick Ssebunya, Barbara Mukanza, Rashidah Nazzinda, Ritah Mbabazi, Abigail Atwine, Juliet Ankunda, Priscilla Kyobutungi, Elizabeth Kaudha, Sharif Musumba, Jesca Nantale, Mariam Naabalamba, Mangadalen Nansaigi, Diana Antonia Rutebarika, Mary Nannungi, Benson Ouma, Edward Bagirigomwa, Eddie Rubanga, Josephine Namusanje, Henry Mugerwa, Crispus Katemba, Deborah Kahinju, Eram David Williams, Juliet Ategeka, Ocitti Sunday Paul Labeja, Joan Nantege, Shirat Nakabiri, Jeremy Kirunda, Charles Draleku, Jamilah Namwanga, Joan Nangiya, Baker Rubinga, Rodney Kazooba, Cate Naluyima, Christopher Lwanga, Faith Balmoi Labote, Dridah Nakiboneka, Bibian Nakigozi Nakato, Christine Nambi, Milly Ndigendawani, Jovia Kavuma, Timothy Lubwama, Frank Mbamanya, Nabuuma Haawa Muweesi, Carol Otike, Mwanje John Bosco, Anna Ritah Namuganga

**University of Zimbabwe Clinical Research Centre, Zimbabwe**: Mutsawashe Bwakura‐Dangarembizi, Kusum Nathoo, Hilda Mujuru, Misheck Phiri, Stuart Chitongo, Wendy Mapfumo, Sandra Musarurwa, Taona Mudzviti, Columbus Moyo, Shepherd Mudzingwa, Shirley Mutsai, Vivian Mumbiro, Joy Chimanzi, Makhosonke Ndlovu, Maureen Tshuma, Ruth Nhema, Ennie Chidziva, Cleopatra Langa, Godfrey Musoro, Dorinda Mukura, Edson Marimo, Tinashe Chidemo, Bernadette Malunda, Musunga Tomu, Lynnet Nyakudya, Nicholas Dhibi, Alfed Kateta, Sidney Sithole, Lynette Chibanda, Deka Vincent, Elaine Mwandiwata, Secrecy Gondo, Moses Chitsamatanga, Farai Selina Matimba, Tsitsi Gwenzi, Nathalie Mudzimirema, Allen Matubu, Prosper Sibonile Dube, Pia Ngwaru, Joyline Bhiri, Trust Mukanganiki, Vinie Kouamou, Rufaro Chivaura, Xeshelihle Mhlanga, Hazel Patricia Munyama, Sibusisiwe Weza, Vongai Margaret Chanaiwa, Shamiso Barbara Gwande, Constantine Mutata, Dorothy Murungu

**Moi University Clinical Research Centre, Kenya**: Abraham Siika, Winstone Nyandiko, Cecilia Kiilu, Charity Wambui, Ronald Tonui, Viola Kirui, Caroline Watiri Maina, Florence Kiwunja Njulu, Cornelius Chege, Natalie Sang, Beatrice K. Jakait, Damaris Lagat, Salinah Cherutich, Benedister Kangogo, Cheruiyot, Brian Odul, Hilda Kaziga, Teri Tarus, Getruth Jerop Kiptoo, Vincent Kipchirchir, Clinton Otieno Achida, Tom Oyoo Osoro, Jairus Kipyego, Violah Samoei, Martha Mokeira Mokaya, Wilson Lokitala Ekiru, Ruth Bosire, Rispher Chepkoech, Festus Kirwa Rugut, Matthew Justus Mutuku, Hellen Jerotich Kiplagat, Millicent Orido, Eslyne Jepkemboi, Vincent Otieno Onyango, Alice Mudogo, Mary Chebet, Joan Chepkurui Kotut, Philemon Kipchirchir Mutai, Eunice Jepkemboi, Julie Chepchumba Choge, Evans Ochieng Ouno, Cecilia Chebet Mengich, Amina Msuo Shali, Solomon Kimurgor Ngetich, Dorcas Akinyi Opot, Shauri Kahindi Gona, Richard Kipsang Kirui, Damaris Jepchumba Kiprutto, Mowlem Pierre, Dorcus Adhiambo, Nicholas Kigen, Sophia Simatwa, Michael Njenga, Cornelius Magut, Kennedy Oyamo, Edna Jepngetich, Gideon Obila, Lydiah Maru, Victor Kibet Langat, Iris Jaluha Sande, Scholastica Njeri Wanjiru, Martha Kaimuri Mwongela

**Durban International Clinical Research Site, Enhancing Care Foundation, South Africa**: Moherndran Archary, Sundrapragasen Pillay, Rosie Mngqibisa, Raziya Bobat, Nozibusiso Rejoice Mosia, Precious Sphiwe Cebekhulu, Nombuso Nkosi, Rashina Nundlal, Saajida Rizvi, Jabu Mkhulise, Innocentia Thandokuhle Mncube, Nathaniel Malatjie, Shingirai Chimene, Zethu Mnyandu, Sheleika Singh, Clement Pillay, Tiyara Arumugam, Shauna Francis, Michelle Moodley, Sylvia Nylobo, Fathima Mather, Ida Mundhree, Popi Shabalala, Ntombizonta Ignatia Phewa, Khombisile Muthwa, Simangele Abigail Bengu, Tshamand Fulufhelo Netshitangani, Rayania Premoutt, Kelly Shermon Clark, Mthokozisi Khuzwayo

**Neuropsychiatric sub‐study**: Cissy Kityo, Henry Mugerwa

**Social Science sub‐studies**: Janet Seeley, Sarah Bernays, Nothando Ngwenya, Tawanda Makusha, Zinhle Ngubo, Phindile Guga, Londiwe Shandu, Tamlyn Seunanden, Stella Namukwaya, Allen Asiimwe, Alice Nassanga

**Health Economics sub‐study**: Paul Revill, Simon Walker

**PK sub‐study**: David Burger, Angela Colbers, Tom Jacobs, Lisanne Bevers

**Youth Trials Board**: Magda Conway, Lungile Jafta, Mercy Shibemba, Carlo Giaquinto

**Trial Steering Committee Members**: Karina Butler, Sam Phiri, Avy Violari, Nathan Ford, Imelda Mahaka, Sarah Pett, Addy Kekitiinwa, Hebron Kalikwani, Priscilla Nakiwala

**Data Monitoring Committee Members**: Anton Pozniak, Rodolphe Thiebaut, Jane Crawley

**Data Monitoring Committee Observers**: Violet Korutaro

## References

[1] S. J. Reynolds , C. Kityo , C. W. Hallahan , et al., “A Randomized, Controlled, Trial of Short Cycle Intermittent Compared to Continuous Antiretroviral Therapy for the Treatment of HIV Infection in Uganda,” PLoS ONE 5, no. 4 (2010): e10307, 10.1371/journal.pone.0010307. PMC2860845 20442758

[2] M. Dybul , T.‐W. Chun , C. Yoder , et al., “Short‐Cycle Structured Intermittent Treatment of Chronic HIV Infection With Highly Active Antiretroviral Therapy: Effects on Virologic, Immunologic, and Toxicity Parameters,” Proceedings of the National Academy of Sciences 98, no. 26 (2001): 15161–15166, 10.1073/pnas.261568398. PMC65000 11734634

[3] C. J. Cohen , A. E. Colson , A. G. Sheble‐Hall , et al., “Pilot Study of a Novel Short‐Cycle Antiretroviral Treatment Interruption Strategy: 48‐Week Results of the Five‐Days‐On, Two‐Days‐Off (FOTO) Study,” HIV Clinical Trials 8, no. 1 (2007): 19–23, 10.1310/hct0801-19. 17434845

[4] P. de Truchis , L. Assoumou , and R. Landman , “Four‐Days‐a‐Week Antiretroviral Maintenance Therapy in Virologically Controlled HIV‐1‐Infected Adults: The ANRS 162–4D Trial,” Journal of Antimicrobial Chemotherapy 73, no. 3 (2017): 738–747. 10.1093/jac/dkx434 29186458

[5] M. Casale , A. Carlqvist , and L. Cluver , “Recent Interventions to Improve Retention in HIV Care and Adherence to Antiretroviral Treatment Among Adolescents and Youth: A Systematic Review,” AIDS Patient Care and STDs 33, no. 6 (2019): 237–252, 10.1089/apc.2018.0320. PMC6588099 31166783

[6] The BREATHER (Penta 16) Trial Group , “Weekends‐Off Efavirenz‐Based Antiretroviral Therapy in HIV‐Infected Children, Adolescents, and Young Adults (BREATHER): A Randomised, Open‐Label, Non‐Inferiority, Phase 2/3 Trial,” Lancet HIV 3, no. 9 (2016): e421–e430, 10.1016/S2352-3018(16)30054-6. PMC4995440 27562743

[7] K. Butler , J. Inshaw , D. Ford , et al., “BREATHER (PENTA 16) Short‐Cycle Therapy (SCT) (5 Days On/2 Days Off) in Young People With Chronic Human Immunodeficiency Virus Infection: An Open, Randomised, Parallel‐Group Phase II/III Trial,” Health Technology Assessment 20, no. 49 (2016): 1–108, 10.3310/hta20490. PMC4947878 27377073

[8] A. Turkova , C. L. Moore , K. Butler , et al., “Weekends‐Off Efavirenz‐Based Antiretroviral Therapy in HIV‐Infected Children, Adolescents and Young Adults (BREATHER): Extended Follow‐Up Results of a Randomised, Open‐Label, Non‐Inferiority Trial,” PLoS ONE 13, no. 4 (2018): e0196239, 10.1371/journal.pone.0196239. PMC5912750 29684092

[9] S. Bernays , S. Paparini , J. Seeley , S. Namukwaya Kihika , D. Gibb , and T. Rhodes , “Qualitative Study of the BREATHER Trial (Short Cycle Antiretroviral Therapy): Is It Acceptable to Young People Living With HIV?,” BMJ Open 7, no. 2 (2017): e012934, 10.1136/bmjopen-2016-012934. PMC5318557 28213595

[10] R. Landman , P. de Truchis , L. Assoumou , et al., “A 4‐Days‐On and 3‐Days‐Off Maintenance Treatment Strategy for Adults With HIV‐1 (ANRS 170 QUATUOR): A Randomised, Open‐Label, Multicentre, Parallel, Non‐Inferiority Trial,” Lancet HIV 9, no. 2 (2022): e79–e90, 10.1016/S2352-3018(21)00300-3. 35120640

[11] D. Luise , E. Lattuada , S. Rizzardo , et al., “Short‐Cycle Therapy in HIV‐Infected Adults: Rilpivirine Combination 4 Days On/3 Days Off Therapy,” Journal of Antimicrobial Chemotherapy 77, no. 3 (2022): 747–752, 10.1093/jac/dkab442. 34849955

[12] B. Hoellinger , D. Rey , P. Klee , et al., “Real‐Life Evaluation of Intermittent Triple Antiretroviral Therapy Maintenance Strategies,” Infectious Diseases Now 55, no. 8 (2025): 105164, 10.1016/j.idnow.2025.105164. 41052650

[13] A. R. Kekitiinwa , A. Jennings , M. Bwakura‐Dangarembizi , et al., “Efficacy and Safety of Short‐Cycle, Dolutegravir‐Based Antiretroviral Therapy in Adolescents Living With HIV (BREATHER Plus): A Multicentre, Open‐Label, Randomised, Parallel, Two‐Arm, Non‐Inferiority Trial,” Lancet HIV 13, no. 6 (2026): e367–e379, 10.1016/S2352-3018(25)00326-1. 41962560

[14] B. E. van Wyk and L.‐A. C. Davids , “Challenges to HIV Treatment Adherence Amongst Adolescents in a Low Socio‐Economic Setting in Cape Town,” Southern African Journal of HIV Medicine 20, no. 1 (2019): 1–7. 10.4102/sajhivmed.v20i1.1002 PMC6852420 31745433

[15] F. Besthorn , E. N. Kalomo , E. Lightfoot , and M. Liao , “The Relationship Between Social Support and Anxiety Amongst Children Living With HIV in Rural Northern Namibia,” African Journal of AIDS Research 17, no. 4 (2018): 293–300, 10.2989/16085906.2018.1534748. 30466364

[16] N. Tarantino , A. Lowery , and L. K. Brown , “Adherence to HIV Care and Associated Health Functioning Among Youth Living With HIV in Sub‐Saharan Africa,” AIDS Review 22, no. 2 (2020): 93–102. 10.24875/AIDSRev.20000101 PMC7517615 32180589

[17] C. Mugo , P. Kohler , M. Kumar , et al., “Effect of HIV Stigma on Depressive Symptoms, Treatment Adherence, and Viral Suppression Among Youth With HIV,” AIDS 37, no. 5 (2023): 813–821, 10.1097/QAD.0000000000003473. PMC10023427 36728652

[18] E. D. Lowenthal , S. Bakeera‐Kitaka , T. Marukutira , J. Chapman , K. Goldrath , and R. A. Ferrand , “Perinatally Acquired HIV Infection in Adolescents From Sub‐Saharan Africa: A Review of Emerging Challenges,” Lancet Infectious Diseases 14, no. 7 (2014): 627–639, 10.1016/S1473-3099(13)70363-3. PMC4074242 24406145

[19] S. MacCarthy , U. Saya , C. Samba , J. Birungi , S. Okoboi , and S. Linnemayr , ““How am I Going to Live?”: Exploring Barriers to ART Adherence Among Adolescents and Young Adults Living With HIV in Uganda,” BMC Public Health [Electronic Resource] 18, no. 1 (2018): 1158, 10.1186/s12889-018-6048-7. PMC6172755 30286746

[20] S. Bernays , J. Lariat , F. Cowan , B. Senzanje , N. Willis , and Z. M. Nenguke , ““They Test My Blood to Know How Much Blood Is in My Body”: The Untapped Potential of Promoting Viral Load Literacy to Support Adherence and Viral Suppression Among Adolescents Living With HIV,” Journal of the International AIDS Society 26, no. 10 (2023): e26153, 10.1002/jia2.26153. PMC10600064 37880186

[21] T. C. Seunanden , N. Ngwenya , S. Namukwaya , et al., “Living in the Shadows: The Persistence of Secrecy in Young People Living With HIV on Antiretroviral Therapy, a Qualitative Study of the BREATHER Plus Trial,” SSM—Qualitative Research in Health 7 (2025): 100560, 10.1016/j.ssmqr.2025.100560.

[22] L. M. Hill , C. E. Golin , A. Pack , et al., “Using Real‐Time Adherence Feedback to Enhance Communication About Adherence to Antiretroviral Therapy: Patient and Clinician Perspectives,” Journal of the Association of Nurses in AIDS Care 31, no. 1 (2020): 25–34, 10.1097/JNC.0000000000000089. PMC6815236 31033629

[23] M. L. Cottrell , T. Hadzic , and A. D. Kashuba , “Clinical Pharmacokinetic, Pharmacodynamic and Drug‐Interaction Profile of the Integrase Inhibitor Dolutegravir,” Clinical Pharmacokinetics 52, no. 11 (2013): 981–994, 10.1007/s40262-013-0093-2. PMC3805712 23824675

[24] B. G. Gazzard , “Efavirenz in the Management of HIV Infection,” International Journal of Clinical Practice 53, no. 1 (1999): 60–64, 10.1111/j.1742-1241.1999.tb11662.x. 10344069

[25] WHO Recommends Dolutegravir as Preferred HIV Treatment Option in all Populations (WHO, 2019).

[26] A. Hill , C. Fairhead , S. Manalu , et al., “Could Reduced Dosing Maintain More People on Antiretrovirals After the Sudden Cuts in USAID Funding? A Crisis Response,” AIDS 39, no. 7 (2025): F1–F4, 10.1097/QAD.0000000000004212. PMC12077339 40265619

